# Alteration of Mammary Gland Development and Gene Expression by *In Utero* Exposure to Cadmium

**DOI:** 10.3390/ijms18091939

**Published:** 2017-09-09

**Authors:** Daniela A. Parodi, Morgan Greenfield, Claire Evans, Anna Chichura, Alexandra Alpaugh, James Williams, Kedra C. Cyrus, Mary Beth Martin

**Affiliations:** 1 Departments of Biochemistry and Molecular & Cellular Biology, Georgetown University, Washington, DC 20007, USA; danielaparodi@yahoo.com (D.A.P.); claire.evans.07@gmail.com (C.E.); 2 Department of Oncology, Georgetown University, Washington, DC 20007, USA; meg63@georgetown.edu (M.G.); amc258@georgetown.edu (A.C.); alpaua@gmail.com (A.A.); jtwilliams@umc.edu (J.W.); kc454@georgetown.edu (K.C.C.); 3 Lombardi Comprehensive Cancer Center, Research Building, 3970 Reservoir Road NW, Washington, DC 20007, USA

**Keywords:** cadmium, estradiol, *in utero*, mammosphere-forming cells, estrogen receptor-α

## Abstract

Environmental exposure to estrogens and estrogen like contaminants during early development is thought to contribute to the risk of developing breast cancer primarily due to an early onset of puberty; however, exposure during key developing windows may also influence the risk of developing the disease. The goal of this study was to ask whether *in utero* exposure to the metalloestrogen cadmium alters mammary gland development due to acceleration of puberty onset or to an effect on early development of the mammary gland. The results show that, in addition to advancing the onset of puberty, *in utero* exposure to the metalloestrogen cadmium altered mammary gland development prior to its effect on puberty onset. *In utero* exposure resulted in an expansion of the number of mammosphere-forming cells in the neonatal mammary gland and an increase in branching, epithelial cells, and density in the prepubertal mammary gland. In the postpubertal mammary gland, there was a further expansion of the mammary stem/progenitor cell population and overexpression of estrogen receptor-alpha (ERα) that was due to the overexpression and altered regulation of the ERα transcripts derived from exons O and OT in response to estradiol. These results suggest that *in utero* exposure to cadmium increases stem/progenitor cells, cell density, and expression of estrogen receptor-alpha that may contribute to the risk of developing breast cancer.

## 1. Introduction

Estrogens are a family of steroidal hormones that promote the growth and differentiation of secondary sex tissues and organs in the female reproductive system [1]. The central role of the hormone has led to the suggestion that the reproductive disorders of animals in the wild and the high incidence of hormone related diseases in children and adults may be due to exposures that mimic the biological effects of estrogens especially if the exposure occurs in early life when the fetus and infant are susceptible to small hormonal changes [2,3]. In Western populations, the onset of puberty has occurred at significantly younger ages. In many European countries, the trend towards earlier puberty onset has ceased [4], but in the United States, it continues especially in African American girls [5]. The onset of puberty, which is triggered by activation of the hypothalamic-pituitary-gonadal axis, is characterized by breast development [6]. During gestation, the mammary gland consists of a small, branched ductal network that does not develop further until puberty [7]. With the onset of puberty, there is significant growth and development of the gland in response to ovarian estrogens and progesterone [8]. There are a number of chemicals in the environment that have estrogen like activity [2] including the naturally occurring phytoestrogens, such as coumestrol and the isoflavone genistein; the synthetic xenoestrogens, such as polybrominated biphenol and bisphenol A; and the metalloestrogens such as the heavy metal cadmium [9,10,11,12,13,14,15]. Soy based formulas containing phytoestrogens [16] and polybrominated biphenol [17] have been linked to the early onset of puberty in epidemiological studies. Animal studies also show that early life exposure to genistein [18], estradiol [19], or cadmium [20] advances the onset of puberty, providing additional evidence that environmental estrogens contribute to early puberty onset.

Cadmium is a metalloestrogen that, similar to estradiol, activates the genomic and nongenomic pathways of ERα [9,10,21,22,23,24,25,26,27] and the membrane estrogen receptor gpr30 [26]. The major source of cadmium contamination in the environment is due to human activities. Discovered in 1817, cadmium was not extensively mined and used in industry [28] until the 1900s with greater than 65% of the world’s production occurring in the last several decades. In the general population, exposure to the metal occurs primarily through dietary sources, cigarette smoking, and, to a lesser degree, drinking water [29,30]. Worldwide, the estimated dietary exposure ranges from 0.12 to 0.49 μg/kg body weight/day with the highest exposure occurring in children 1–6 years of age [29,30,31,32,33,34,35]. In infants 0–1 years of age, the estimated exposure ranges from 0.37 to 2.2 μg/kg body weight (bw)/day [36,37] exceeding the World Health Organization (WHO) Provisional Weekly Intake (PTWI) established for adults. The higher exposure in infants is due to high concentrations of cadmium in formulas and in colostrums and breast milk [36,38,39,40] as a result of the ability of the mammary gland to concentrate the metal [41]. During fetal development, exposure is also significant; cadmium is also easily transferred through the placenta from the mother to the fetus [42]. A few epidemiological studies have found a link between occupational and nonoccupational exposure to cadmium and an increased risk of developing breast cancer. The first study, a hypothesis-generating case-control study that examined death certificates coded for occupation and industry, showed that cadmium was associated with an increased risk of developing the disease [43]. The second study, a retrospective cohort study of women employed as metal platers and coaters also showed that exposure to cadmium was associated with an increased risk of developing breast cancer [44]. In nonoccupationally exposed women, two population based case-control studies also showed an association between cadmium and an increased risk of developing breast cancer [45,46]. Although these epidemiological studies suggest an association between cadmium and breast cancer, the studies do not establish a cause and effect relationship between the metal and the disease. We have previously shown that environmentally relevant amounts of cadmium have potent estrogen like activity in vivo and that *in utero* exposure to the metal advances the onset of puberty and alters development of the pubertal mammary gland. In the pubertal mammary gland, there is an increase in the parenchymal area and number of terminal end buds and a decrease in the number of alveolar buds [10,20]. In this study, we asked whether the alterations in the pubertal mammary gland were due to the advancement of puberty onset or to an effect on the early formation of the gland. The results show that *in utero* exposure to cadmium altered the development of the mammary gland prior to its effect on the hypothalamic-pituitary-gonadal axis. *In utero* exposure to the metal caused an expansion of the mammosphere-forming cell population and an increase in the expression of genes that influence the development and response of the mammary gland to ovarian hormones, which may contribute to the risk of developing breast cancer.

## 2. Results

### 2.1. Effects of Early Life Exposure to Cadmium on Vaginal Opening in Female Offspring

**Figure 1:**
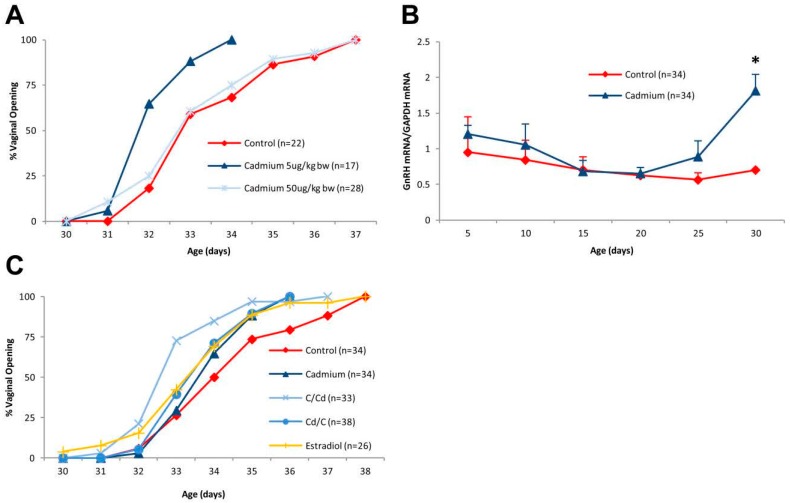
Effects of early life exposure to cadmium on puberty onset in female offspring. Pregnant female rats were treated with cadmium (5 or 50 μg/kg bw) by i.p. injection on days 12 and 17 of gestation and the female offspring were examined. (**A**) Effect of early life exposure to cadmium on the time of vaginal opening. Pregnant female rats were treated with cadmium (5 or 50 μg/kg bw) and the female offspring were monitored for vaginal opening (*n* = 17–28 offspring/group); (**B**) effect of early life exposure to cadmium on the expression of GnRH in the hypothalamus. Pregnant rats were treated with cadmium (5 μg/kg bw) and the expression of GnRH in the female offspring was determined on postnatal days 0, 5, 10, 15, 20, 25, and 30. The amount of GnRH mRNA was determined by a qRT-PCR assay and normalized to the amount of glyceraldehyde 3-phosphate dehydrogenase (GAPDH) mRNA (mean ± SEM; *n* = 2–3 offspring/group; * *p* < 0.05 for treatment groups with *n* = 3); (**C**) effect of exposure to cadmium while *in utero* and/or during postnatal development on the time of vaginal opening. Pregnant female rats were treated with cadmium (5 μg/kg bw) or ethinyl estradiol. Female offspring were crossfostered at birth and monitored for vaginal opening. Cadmium treated animals were exposed to cadmium while *in utero* and during lactation; C/Cd treated animals were exposed to cadmium during lactation; Cd/C treated animals were exposed to cadmium while *in utero* (mean ± SEM; *n* = 26–38 offspring/group; * *p* < 0.05).

We have previously shown that early life exposure to doses of cadmium (0.5 or 5 μg/kg body weight), which are similar to or lower than the WHO PTWI, induce an earlier onset of vaginal opening in female offspring [20]. To ask whether a higher dose of the metal has a greater effect on the time of vaginal opening, pregnant animals were treated with cadmium (5 or 50 μg/kg bw) (Figure 1A). Interestingly, there was no significant difference in vaginal opening between control animals (postnatal day 33.77 ± 0.32) and animals exposed *in utero* to the higher dose of cadmium (50 μg/kg bw; postnatal day 33.46 ± 0.30). However, in animals exposed to the lower dose of the metal (5 μg/kg bw), vaginal opening occurred significantly earlier (postnatal day 32.41 ± 0.19; *p* < 0.05) and was not due to an increase in body weight (data not shown). As vaginal opening is controlled by the reactivation of the gondotrophin releasing hormone (GnRH) pulse generator [47], the amount of GnRH mRNA in the hypothalamus was measured on postnatal days 5, 10, 15, 20, 25 and 30 (Figure 1B). In the control animals, GnRH mRNA began to increase between postnatal days 25 and 30 [48]. In the animals exposed to cadmium (5 μg/kg bw), GnRH mRNA began to increase between postnatal days 20 and 25 and was significantly higher on postnatal 30, suggesting that early life exposure to cadmium alters the expression of GnRH. To ask whether the timing of exposure to the metal influenced the onset of puberty, female offspring exposed to the lower dose of cadmium and crossfostered at birth, i.e., pups exposed to cadmium while *in utero*, were crossfostered with control dams and control pups were crossfostered with cadmium treated dams (Figure 1C). In control animals, vaginal opening occurred on postnatal day 34.76 ± 0.30 [48]. In animals exposed to cadmium while *in utero* and fostered with either a control dam or a cadmium treated dam, vaginal opening occurred earlier (postnatal day 33.24 ± 0.20 and 34.15 ± 0.18, respectively; *p* < 0.05). In control animals fostered with cadmium treated dams, vaginal opening also occurred earlier (33.95 ± 0.18, *p* < 0.05). Early onset of vaginal opening was not due to an increase in body weight (data not shown). Taken together, the results show that exposure to a low dose of cadmium while *in utero* or during neonatal development accelerates vaginal opening.

### 2.2. Effects of In Utero Exposure to Cadmium on the Morphology of the Mammary Gland of Female Offspring

**Figure 2:**
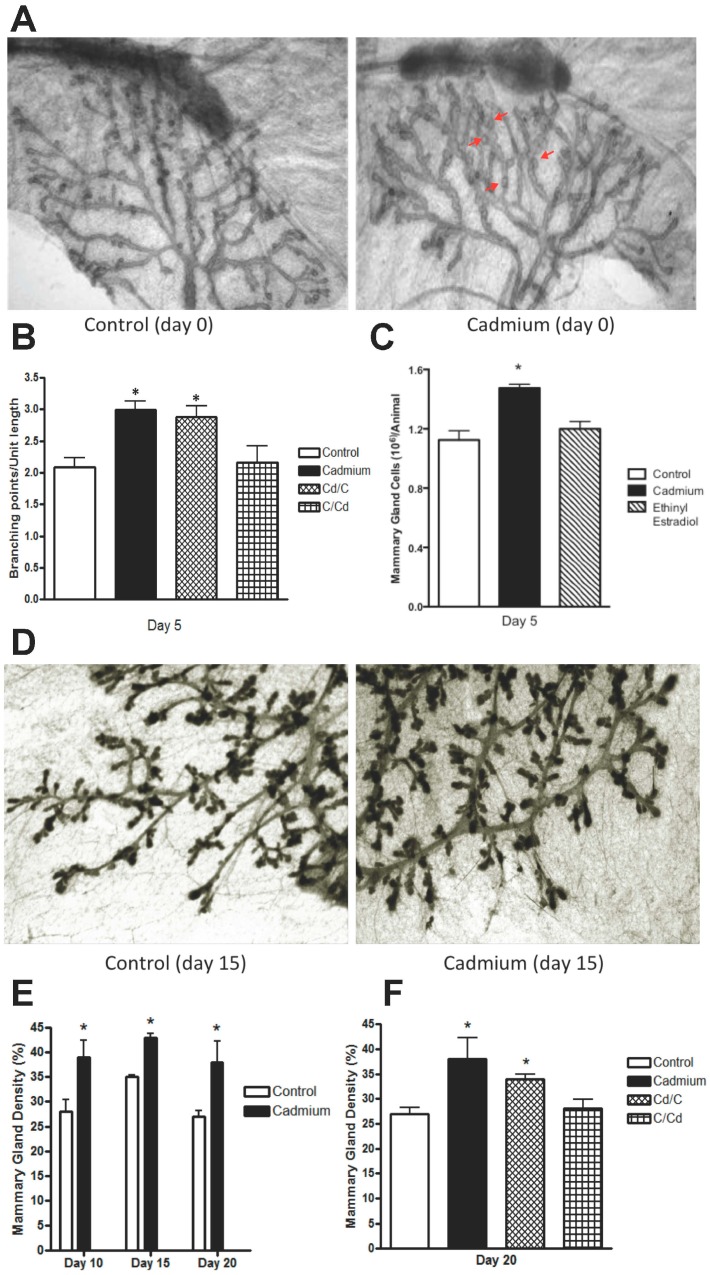
Effects of *in utero* exposure to cadmium on the morphology of the mammary gland of female offspring. Pregnant female rats were treated with cadmium (5 μg/kg bw) or ethinyl estradiol (50 μg/kg bw) and the female offspring were crossfostered as described in Figure 1. Cadmium treated animals were exposed to cadmium while *in utero* and during lactation; Cd/C treated animals were exposed to cadmium while *in utero*; and C/Cd treated animals were exposed to cadmium during lactation. For morphological analyses, the fourth abdominal mammary glands were excised. (**A**) *In utero* effects on mammary gland morphology. Digital image on postnatal day 0 from a control animal (representative image) or an animal exposed to cadmium (representative image). Arrows indicate abnormal lateral budding; (**B**) *in utero* effects on mammary gland branching. The number of branch points was quantified on postnatal day 5 as branches per unit length (mean ± SEM; *n* = 3 offspring/group; * *p* < 0.05); (**C**) *in utero* effects on the number of epithelial cells in the mammary gland. Both fourth abdominal mammary glands were excised on postnatal day 5. Epithelial cells were isolated and counted (mean ± SEM; *n* = 4 offspring/control group, *n* = 3 offspring/cadmium group, *n* = 2 offspring/ethinyl estradiol group; * *p* < 0.05 for control vs. cadmium). (**D**–**F**) *In utero* effects on mammary gland density on postnatal days 10, 15, and 20; (**D**) digital image from 15-day-old control animal (representative image) or animal exposed to cadmium (representative image); (**E**,**F**) mammary gland density is expressed as the percent of epithelium in the fat pad (mean ± SEM; *n* = 3 offspring/group; * *p* < 0.05). Digital images of control offspring on postnatal days 0 and 15 were adapted with permission from Reproductive Toxicololgy [48].

Our previous studies show that early life exposure to cadmium also increases the parenchymal area and number of terminal end buds and decreases the number of alveolar buds in the pubertal mammary gland. To ask whether these alterations are due to the acceleration of puberty onset or to an effect on the early formation of the mammary gland, pregnant animals were again treated with cadmium (5 μg/kg bw) and the female offspring were crossfostered. On postnatal days 0, 5, 10, 15 and 20, the mammary gland architecture was examined (Figure 2). An abnormal pattern of lateral budding [49] along the ducts of the glands was observed in animals exposed while *in utero* to cadmium (Figure 2A). The number of branch points along the ducts was counted in the 5-day-old animals to determine whether exposure to the metal alters branching in the gland (Figure 2B). There was no difference in the number of branch points in the mammary glands of control animals fostered with control dams (2.08 ± 0.26 branch points/unit length [48]) compared to the number of branch points in the glands of control animals fostered with cadmium treated dams (2.16 ± 0.4 branch points/unit length). However, the number of branch points was significantly higher in the mammary glands of animals exposed to cadmium while *in utero* and fostered with either control dams (2.88 ± 0.30 branch points/unit length; *p* < 0.05) or cadmium treated dams (2.99 ± 0.19 branch points/unit length; *p* < 0.05) suggesting that *in utero* exposure to cadmium increases branching in the gland. To determine whether the increase in branching leads to an increase in the number of epithelial cells, the cells were isolated and counted (Figure 2C). For comparison, the results from animals exposed *in utero* to ethinyl estradiol (50 μg/kg bw/day) are included (Figure 2C; [48]). In animals exposed *in utero* to cadmium, there was a significant 1.31-fold increase (*p* < 0.05) in the number of epithelial cells. To ask whether the increase in epithelial cells persists throughout the prepubertal development of the gland, the density of the mammary gland was measured on postnatal days 10, 15, and 20 (Figure 2D–F). Compared to controls (28%, 35% and 27%, respectively [48]), there was a significant increase (*p* < 0.05) in mammary gland density (39%, 43% and 38%, respectively) in animals exposed to cadmium. To establish that the increase in density is due to an increase in branching, mammary density was measured in the crossfostered animals on postnatal day 20. Consistent with the changes in branching, there was an increase in density in animals exposed to cadmium while *in utero* (34%, *p* < 0.05) and no increase in density in control animals fostered with cadmium treated dams (28%). Taken together, the results demonstrate that prior to the onset of puberty, there is a consistent 30% to 40% increase in the number of branch points, number of epithelial cells, and density in the mammary glands of animals exposed to cadmium while *in utero*, but no increase in control animals fostered with cadmium treated dams, suggesting that exposure to the metal while *in utero* affects the early formation of the mammary gland.

### 2.3. Effects of In Utero Exposure to Cadmium on Mammosphere-Forming Cells in the Mammary Glands of Female Offspring

**Figure 3:**
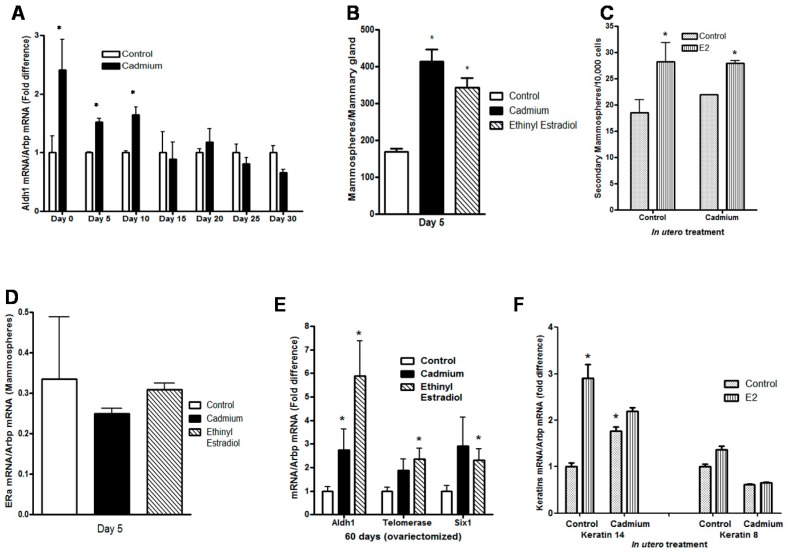
Effects of *in utero* exposure to cadmium on mammosphere-forming cells in the mammary gland of female offspring. Pregnant female rats were treated with cadmium (5 μg/kg bw) or ethinyl estradiol (50 μg/kg bw) as described in Figure 1. (**A**) *In utero* effects on Aldh1 expression in the prepubertal mammary gland. Aldh1 mRNA was measured on postnatal days 0, 5, 10, 15, 20, 25, and 30 by a qRT-PCR assay, normalized to Arbp mRNA, and presented as fold difference (mean ± SEM; *n* = 2–3 offspring/group; * *p* < 0.05); (**B**) *in utero* effects on the number of mammospheres obtained from female offspring on postnatal day 5 (mean ± SEM; *n* = 4 offspring/group; * *p* < 0.05); (**C**) *in utero* effects on estradiol induced proliferation of mammospheres. First generation mammospheres were digested with trypsin, stem/progenitor (S/P) cells were selected in serum-free media under non-adherent conditions in the presence or absence of 17β-estradiol (1 nM), and the second generation mammospheres were counted (mean ± SEM; *n* = 2 offspring/group); (**D**) *in utero* effects on ERα expression in mammospheres obtained from female offspring on postnatal day 5. ERα mRNA was determined by a qRT-PCR assay and normalized to Arbp mRNA (mean ± SEM; *n* = 2 offspring/group); (**E**) *in utero* effects on stem cell markers in the adult mammary gland. Female offspring were ovariectomized on postnatal day 45. On postnatal day 60, Aldh1, telomerase, and Six1 mRNA were measured by a qRT-PCR assay, normalized to Arbp mRNA, and presented as fold difference (mean ± SEM; *n* = 8–10 offspring/group; * *p* < 0.05); (**F**) *in utero* effects on epithelial cell markers in the adult mammary gland. Female offspring were ovariectomized on postnatal day 45. Beginning on postnatal day 56, the animals were treated daily with 17β-estradiol. On postnatal day 60, keratin 14 and keratin 8 mRNA were measured by a qRT-PCR assay, normalized to Arbp mRNA, and presented as fold difference (mean ± SEM; *n* = 8–10 offspring/group; * *p* < 0.05).

As *in utero* exposure to cadmium increased the number of branch points in the neonatal mammary gland, the effects of exposure on the mammary stem and progenitor cell population [50] was investigated. In the mammary gland, aldehyde dehydrogenase 1A1 (Aldh1A1), a marker of stem/progenitor cells, is expressed at branch points and functions in the proliferation of progenitor cells and branching morphogenesis [51]. In animals exposed *in utero* to cadmium, there was a significant increase in Aldh1A1 mRNA on postnatal days 0, 5, and 10 (2.41-, 1.52-, and 1.65-fold increase, respectively; *p* < 0.05) but no difference on postnatal days 15 through 30 (Figure 3A). Mammary stem cells can be partially characterized by their ability to form spheroids when grown in nonadherent conditions [52,53]. Mammospheres [54,55] were isolated from the glands of 5-day-old offspring (Figure 3B) to ask whether there was also an increase in the number of mammosphere-forming cells. Compared to control animals, there was a 2.44-fold increase in the number of mammospheres derived from the mammary glands of animals exposed *in utero* to cadmium. For comparison, the results from animals exposed *in utero* to ethinyl estradiol are also shown (Figure 3). To determine whether the mammospheres had stem-like properties, the potential for self-renewal was tested by subculturing the mammospheres every 10 days. The mammospheres produced secondary and tertiary generation mammospheres with an efficiency of approximately 2/1000 cells (Figure 3C). The mammospheres expressed Aldh1A1 [56] and CD24 [57] (data not shown). The mammospheres also expressed estrogen receptor-alpha (ERα) [58,59,60]. Mammospheres isolated from control or exposed animals expressed similar amounts of ERα (Figure 3D). When treated with 17β-estradiol, there was an increase in the number of mammospheres derived from control or cadmium exposed animals (1.53- and 1.3-fold increase, respectively; *p* < 0.05; Figure 3C). To ask whether *in utero* exposure to the metal also affected the postpubertal mammary gland, the expression of Aldh1A1, telomerase [61,62], and Six1 [63,64,65], genes that are associated with stem and progenitor cells, was determined on postnatal day 60. To avoid the effects of estradiol on gene expression, the animals were ovariectomized on postnatal day 45 (Figure 3E) [66]. In the adult animals exposed *in utero* to cadmium, there was a significant increase in Aldh1A1 mRNA (2.74-fold increase; *p* < 0.05) as well as an increase in telomerase mRNA (1.9-fold; *p* = 0.057) and Six1 mRNA (2.9-; *p* = 0.075). To ask whether *in utero* exposure altered the types of epithelial cells, the expression of keratin 14, a basal cell marker, and keratin 8, a luminal cell marker, was determined (Figure 3F). In animals exposed *in utero* to cadmium, there was a significant increase in keratin 14 mRNA (1.77-fold; *p* < 0.05) but no effect on keratin 8 mRNA. Treatment with 17β-estradiol had no effect on keratin 14 or 8 mRNA in animals exposed *in utero* to cadmium but increased keratin 14 mRNA in control animals (Figure 3F). The increase in the number of mammospheres together with the increase in Aldh1A1 and keratin 14 mRNAs suggests that *in utero* exposure to cadmium causes an expansion of the mammosphere-forming cells and an increase in the basal cell population in the mammary gland.

### 2.4. Effects of In Utero Exposure to Cadmium on Gene Expression in the Mammary Gland of Female Offspring

**Figure 4:**
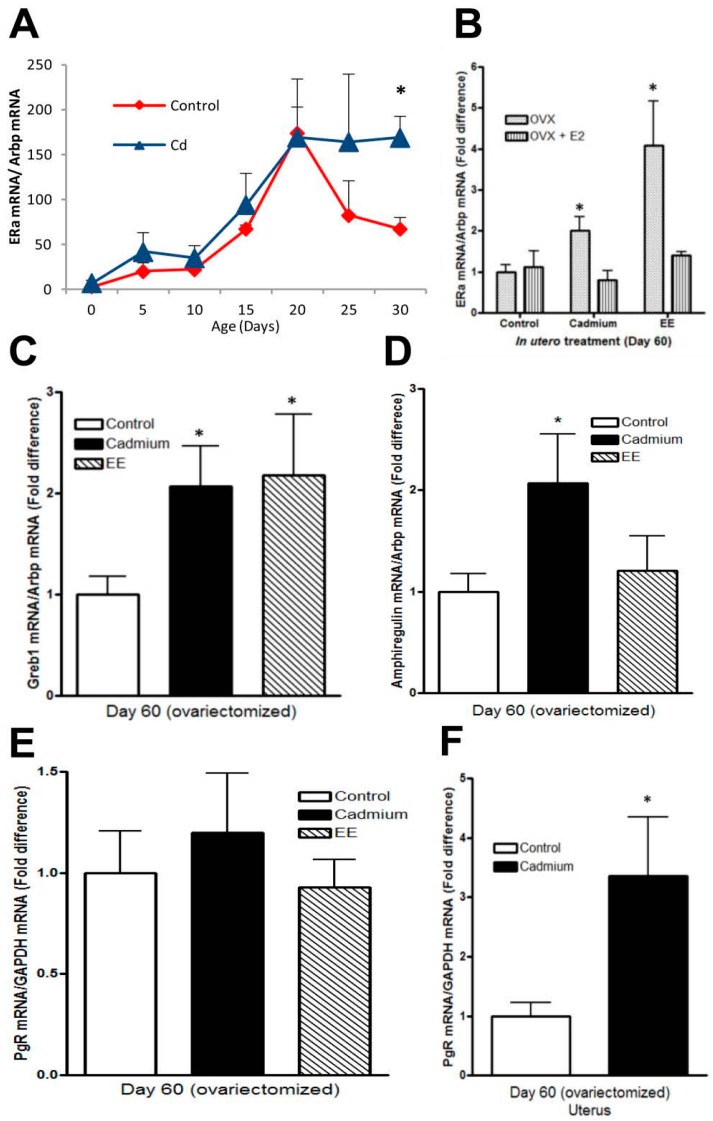
Effects of *in utero* exposure to cadmium on gene expression of ERα in the female offspring. Pregnant female rats were treated with cadmium (5 μg/kg bw) or ethinyl estradiol (50 μg/kg bw) as described in Figure 1 and the female offspring were examined. (**A**) *In utero* effects on ERα expression in the prepubertal mammary gland. The amount of ERα mRNA was measured on postnatal days 0, 5, 10, 15, 20, 25, and 30 by qRT-PCR assay and normalized to Arbp mRNA (mean ± SEM; *n* = 3–11 offspring/group; * *p* < 0.05); (**B**) *in utero* effects on the regulation of ERα in the adult mammary gland. Female offspring were ovariectomized on postnatal day 45. Beginning on postnatal day 56, the animals were treated daily with 17β-estradiol. On postnatal day 60, the amount of ERα mRNA was measured by a qRT-PCR assay and normalized to Arbp mRNA. Data are presented as fold difference (mean ± SEM; *n* = 7–11 offspring/group; * *p* < 0.05); (**C**–**E**) *in utero* effects on gene expression in the adult mammary gland. Female offspring were ovariectomized on postnatal day 45. On postnatal day 60, the amount of Greb1 (**C**), amphiregulin (**D**), and PgR (**E**) mRNA was measured by a qRT-PCR assay, normalized to Arbp mRNA, and presented as fold difference (mean ± SEM; *n* = 7–11 offspring/group; * *p* < 0.05); (**F**) *in utero* effects on progesterone receptor expression in the adult uterus. Female offspring were ovariectomized on postnatal day 45. On postnatal day 60, uteri were excised and the amount of PgR mRNA was measured by a qRT-PCR assay, normalized to GAPDH mRNA, and presented as fold difference (mean ± SEM; *n* = 7–11 offspring/group; * *p* < 0.05).

Overexpression of ERα is thought to be an initial event in the development of ER positive breast cancer and *in utero* exposure to endocrine disrupting chemicals has been shown to alter expression of ERα in the mammary glands of exposed offspring [67,68,69,70]. To determine whether *in utero* exposure to cadmium also alters ERα expression, ERα mRNA in the mammary gland was measured on postnatal days 0, 5, 10, 15, 20, 25 and 30 and on postnatal day 60 in animals that were ovariectomized on postnatal day 45 (Figure 4A,B). In control animals, the expression of ERα mRNA increased from postnatal day 0 to day 20 and decreased on postnatal day 25 and day 30 [48]. In animals exposed *in utero* to cadmium, the expression of ERα mRNA also increased from postnatal day 0 to day 20 but remained elevated on postnatal days 25 and 30 (Figure 4A) and on postnatal day 60 (Figure 4B). In the latter animals, *in utero* exposure to cadmium resulted in a 2.0-fold increase (*p* < 0.05) in ERα mRNA. In Sprague–Dawley rats, ERα is expressed in the luminal epithelial cells [71,72]. The increase in ERα mRNA but no change in keratin 8 (Figure 3F and Figure 4B) suggests that *in utero* exposure to the metal results in the overexpression of ERα in the luminal cells of the postpubertal mammary gland. In breast cancer cells, ERα is overexpressed and downregulated by 17β-estradiol [73,74,75]. To determine whether *in utero* exposure to cadmium also alters the hormonal regulation of ERα, the ovariectomized animals were then treated with 17β-estradiol. Treatment with 17β-estradiol had no effect on the expression of ERα mRNA in control animals but decreased ERα mRNA in animals exposed *in utero* to cadmium (Figure 4B). The decrease in ERα mRNA but no change in the basal and luminal cell markers in the cadmium exposed offspring following treatment with 17β-estradiol (Figure 3F and Figure 4B) suggests that, in addition to altering the expression of ERα, *in utero* exposure to cadmium alters the hormonal regulation of the receptor in the postpubertal mammary gland.

To ask whether *in utero* exposure to cadmium alters the expression of genes that are regulated by estradiol or involved in mammary gland development, growth, and tumorigenesis, candidate genes were measured in the mammary gland and uterus of the ovariectomized animals (Figure 4C–F). *In utero* exposure to cadmium increased the expression of GREB1, an ERα co-factor [76,77], and ampiregulin, a growth factor that mediates the effects of estrogen on ductal elongation [78,79,80,81], but had no effect on the expression of progesterone receptor (PgR) mRNA in the mammary gland. In contrast to the mammary gland, *in utero* exposure to the metal increased the expression of PgR mRNA in the uterus but had no effect on the expression of GREB1 and amphiregulin (data not shown).

### 2.5. Effects of In Utero Exposure to Cadmium on the Expression of ERα Transcripts in the Mammary Gland of Female Offspring

**Figure 5:**
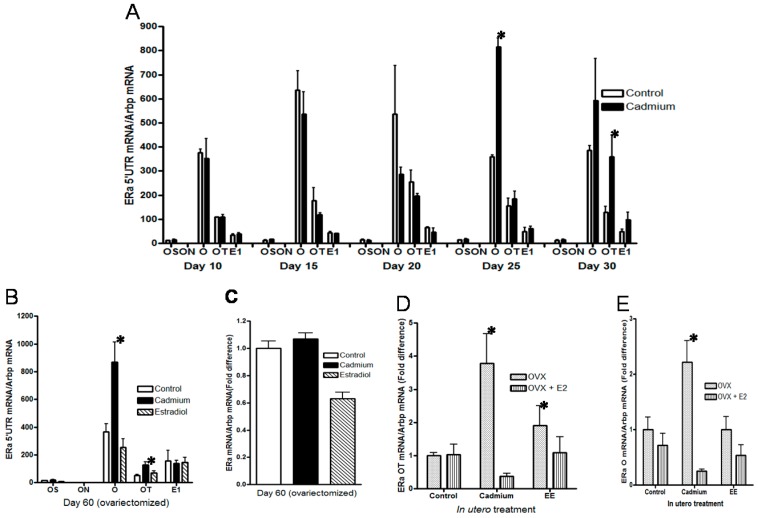
Effects of *in utero* exposure to cadmium on the expression of ERα transcripts in the mammary gland of female offspring. Pregnant female rats were treated with cadmium (5 μg/kg bw) or ethinyl estradiol (50 μg/kg bw) as described in Figure 1 and the female offspring were examined. (**A**) *in utero* effects on ERα transcripts in the prepubertal mammary gland. The amount of the5’ untranslated RNA (5′UTR) ERα mRNA was measured on postnatal days 10, 15, 20, 25, and 30 by qRT-PCR and normalized to Arbp mRNA (mean ± SEM; *n* = 2–3 offspring/group; * *p* < 0.05); (**B**) *in utero* effects on ERα transcripts in the adult mammary gland. Female offspring were ovariectomized on postnatal day 45. On postnatal day 60, the amount of ERα 5′UTR transcript was measured by a qRT-PCR assay and normalized to Arbp mRNA (mean ± SEM; *n* = 7–12 offspring/group; * *p* < 0.05); (**C**) *in utero* effects on ERα gene transcription in the adult mammary gland. Female offspring were ovariectomized on postnatal day 45, nuclei were isolated on postnatal day 60, and a nuclear run on assay was performed. The amount of the nascent ERα transcript was measured by qRT-PCR, normalized to Arbp transcript, and presented as fold difference (*n* = 8–10 offspring); (**D**,**E**) *in utero* effects on the regulation of ERα transcripts in the adult mammary gland. Female offspring were ovariectomized on postnatal day 45 and treated for four days with 17β-estradiol beginning on postnatal day 56. On postnatal day 60, the amount of O and OT transcripts was measured by a qRT-PCR assay, normalized to Arbp mRNA, and presented as fold difference (mean ± SEM; *n* = 7–11 offspring/group; * *p* < 0.05).

The human ERα gene has eight promoters [82] and is overexpressed in breast cancer due, in part, to an increase in transcripts transcribed from specific promoters [83,84,85]. The rat ERα gene has five promoters (OS, ON, O, OT, and E1) [86,87,88,89] that result in ERα transcripts with different 5′ untranslated regions (5′UTR). To determine whether *in utero* exposure alters the expression of specific ERα transcripts, the 5′UTRs of ERα mRNA were measured using primers for exons OS, ON, O, OT, and E1 (Figure 5) on postnatal days 10 to day 30 (Figure 5A) and in the ovariectomized animals on postnatal day 60 (Figure 5B). On postnatal days 10, 15 and 20, there was no difference in ERα transcripts between control and exposed animals. However, on postnatal day 25, there was a significant increase in the transcript derived from exon O in the exposed animals (2.28-fold; *p* < 0.05) which remained elevated on postnatal day 30 (2.1-fold increase). On postnatal day 30, there was also a significant increase in the transcript derived from exon OT (2.77-fold; *p* < 0.05). On postnatal day 60, transcripts derived from exons O and OT remained elevated in the exposed animals (2.37- and 2.43-fold; *p* < 0.05, respectively; Figure 5B). To ask whether there was a difference in transcription, a nuclear run on assay was performed. The results showed no difference in transcription of the ERα gene between control animals and animals exposed *in utero* to cadmium (Figure 5C). To ask whether *in utero* exposure also alters the regulation of the O and OT transcripts by estradiol, the ovariectomized animals were then treated with 17β-estradiol (Figure 5D,E). Treatment with 17β-estradiol decreased the expression of the O and OT transcripts in animals exposed to cadmium while *in utero* but had no effect on the expression of the O and OT transcripts in control animals. Taken together, the data suggest that *in utero* exposure to cadmium results in the overexpression and altered the hormonal regulation of specific ERα transcripts in the postpubertal mammary gland of the exposed animals.

## 3. Discussion

Early onset of puberty is thought to contribute to the risk of developing breast cancer due to an increase in lifetime exposure to estrogens. There is also evidence that exposure of the mammary gland to estrogens and estrogen like chemicals during its early formation alters the development of the gland and predisposes it to carcinogenesis. The results of this study show that, in addition to advancing the onset of puberty, *in utero* exposure to cadmium altered mammary gland development in the exposed offspring prior to its effect on puberty onset. In the prepubertal mammary gland, *in utero* exposure to cadmium resulted in an expansion of the mammosphere-forming cell population and an increase in the number of branch points, number of epithelial cells, and density of the mammary gland prior to its effect on the hypothalamic-pituitary-gonadal axis and vaginal opening. In the postpubertal mammary gland, *in utero* exposure resulted in a further expansion of the mammary stem/progenitor cell population, an increase in basal cells, and an increase in the expression of ERα, the ERα cofactor GREB1, and the growth factor amphiregulin; genes that are associated with the growth and response of the mammary gland to ovarian hormones.

Over the past several decades, the global incidence rate of breast cancer has increased [90,91,92], yet the underlying causes of the disease are largely unknown. The most prominent known risk factors for developing breast cancer are associated with lifetime exposure to ovarian hormones and include early menarche [93,94], late menopause [95,96], and postmenopausal use of estrogens and progestins [97,98,99]. In addition to lifetime exposure, there is epidemiological and experimental evidence to suggest that exposure to estrogens during critical windows of development increases the risk of developing breast cancer. An increased risk of developing breast cancer is associated with increased exposure to estrogens during pregnancy, whereas a lower risk of developing the disease is associated with decreased exposure to estrogens [98,100,101,102,103,104,105,106]. Animal studies show that female animals exposed *in utero* to elevated estrogens through the maternal administration of estradiol, diethylstilbesterol (DES), or the phytoestrogen genistein experience an earlier onset of vaginal opening and have a significantly increased risk of developing mammary tumors [18,107,108], supporting the idea that the increased risk of developing breast cancer is due to the longer lifetime exposure to estrogens associated with early puberty. *In utero* exposure to cadmium also advances the onset of vaginal opening in female offspring [20], suggesting that *in utero* exposure to the metal may increase the risk of developing breast cancer due to longer lifetime exposure to estrogens. The present study shows that, that in addition to earlier vaginal opening, *in utero* exposure to the metal alters early development of the mammary gland. The mammary gland is unusual in that it develops and grows throughout the lifetime of a female beginning in fetal life and ending following the first full term pregnancy and lactation [109]. During fetal development and pregnancy, expansion of the mammary stem cell population is important for the development of branches and the elongation of the ducts [110]. In the developing mammary gland, stem cells are located along the ducts [50] and in the terminal end buds [111] and generate multipotent and unipotent progenitor cells that proliferate and differentiate into luminal and basal cells resulting in the development of branches and the elongation of the ducts. In the pregnant mammary gland, stem cell expansion is necessary for the formation and growth of tertiary ducts [112,113,114,115]. In the cadmium exposed offspring, the increase in mammosphere-forming cells in the neonatal mammary gland and the subsequent increase in branches, epithelial cells, and epithelial density in the prepubertal mammary gland suggests that *in utero* exposure to the metal results in an inappropriate expansion of the stem/progenitor cell population during the early development of the mammary gland. The increase in the mammary stem/progenitor cell markers and the overexpression of ERα and GREB1 in the postpubertal mammary gland suggests that *in utero* exposure to the metal also alters the response of the nonpregnant mammary gland to ovarian hormones resulting in the further expansion of the stem/progenitor cells, the putative targets of malignant transformation in the breast.

One of the initial events in the development of ER positive breast cancer is thought to be the overexpression of ERα. The human ERα gene has eight promoters giving transcripts that contain exons F, E1/E2, T1/T2, D, C(2), B, and A [82]. In normal human breast tissue, the 5′UTRs of the ERα transcripts are predominantly from exons C and A with the expression of transcript with exon A greater than the transcript with exon C. In human breast tumors and breast cancer cells, transcripts with exons C, B, and A are overexpressed with the expression of the transcript with exon C greater than the transcript with exon A [83,84,85]. The overexpression of ERα transcripts is due, in part, to changes in the methylation of the ERα promoters [116]. *In utero* exposure to cadmium caused a similar overexpression of ERα in the mammary gland. The rat ERα gene has five promoters resulting in transcripts with 5′UTRs derived from exons OS, ON, O, OT, and E1 [86,87,88,89]. Exons O and OT in the rat are homologous to exons C and B in human, respectively. Similar to the overexpression of the transcripts derived from exons C and B in human breast cancer, *in utero* exposure to cadmium resulted in the overexpression of exons O and OT transcripts in the rat mammary gland. *In utero* exposure to the metal also altered the hormonal regulation of ERα expression. Taken together, the results show that *in utero* exposure to cadmium alters the expression of ERα consistent with the alterations observed in ER positive breast cancer [73].

Although this study was not designed to rigorously compare the effects of cadmium and ethinyl estradiol and should be cautiously interpreted, the results show that *in utero* exposure to cadmium and ethinyl estradiol have similar as well as different effects in the female offspring. Similar to ethinyl estradiol, *in utero* exposure to cadmium accelerated vaginal opening, increased the mammary stem/progenitor cell population, and increased the expression of ERα and the ERα cofactor GREB1. In contrast to ethinyl estradiol, exposure to the metal increased the number of epithelial cells in the prepubertal mammary gland and the expression of amphiregulin in the adult mammary gland suggesting that *in utero* exposure to cadmium alters additional pathways. The mechanism(s) by which cadmium increased the number of epithelial cells and the expression of amphiregulin is not known but may be due to the ability of cadmium to mimic calcium. Cadmium has been shown to activate calcium signal transduction pathways leading to an increase in gene expression and cell proliferation [117]. Alternatively, the effects of cadmium may be mediated through the heavy metal response element [118]. To understand the mechanism(s) by *in utero* exposure to cadmium alters mammary gland development, additional experiments are required.

As mammary stem and/or progenitor cells are thought to be the targets of malignant transformation in the breast [119] and overexpression of ERα is thought to be the first event in the development of ER positive breast cancers, the ability of *in utero* exposure to cadmium to cause an expansion in the mammary stem/progenitor cell population and an increase in the expression of ERα suggests that *in utero* exposure to the metal may predispose the mammary gland to malignant transformation. The increasing prevalence of cadmium in the environment over the last century and its ability to cross the placenta and accumulate in the mammary gland further suggests that exposure to cadmium during early development contributes to the risk of developing breast cancer.

## 4. Materials and Methods

### 4.1. Animals

All animal studies were approved by the Georgetown University Animal Care and Use Committee, Protocol Number 15-047-100247, Approval Date 11/5/2015. Pregnant Sprague–Dawley rats were obtained from Harlan Breeding Facilities (Frederick, MD, USA) on day 7 of gestation and placed on a purified phytoestrogen-free diet that was not supplemented with Cu, Cr and Se (Tekland Lab Animal Diets, Madison, WI, USA, TD02373). In rodents, mammary gland development begins on days 12 to 14 days of gestation. In this study, pregnant female rats were treated with sterile water by i.p. injection on days 12 and 17 of gestation or 5 μg/kg bw of cadmium chloride (Sigma, St. Louis, MI, UAS) dissolved in sterile water by i.p. injection on days 12 and 17 of gestation to mimic the WHO PTWI of 7 μg/kg bw/week. For comparison, pregnant animals were also treated with 50 μg/kg bw of ethinyl estradiol [120] by daily oral gavage starting on day 12 of gestation until birth. For each experiment, the control, cadmium, and ethinyl estradiol exposed animals were treated at the same time. To minimize the number of animals, some, but not all, experiments were conducted simultaneously with another study and shared controls and ethinyl estradiol treated animals. Because some studies shared control and ethinyl estradiol treated animals, some control and ethinyl estradiol values are reported elsewhere [48]. Two days after birth, female pups from each group were pooled and randomly assigned to a dam from that group. Male pups were euthanized. To monitor normal development, eye lid opening and weekly weights were determined. Vaginal opening was monitored daily from postnatal day 25 to 40 and all pups were included in the analysis. The morphological studies were blinded to experimental treatment. Experimental animal groups included pups not exposed *in utero* to cadmium and fostered with control dams, pups not exposed *in utero* to cadmium and fostered with dams exposed to cadmium, pups exposed *in utero* to cadmium and fostered with control dams, pups exposed *in utero* to cadmium and fostered with dams exposed to cadmium, and pups exposed *in utero* to ethinyl estradiol and fostered with dams exposed to ethinyl estradiol. For the vaginal opening and adult ovariectomy studies, two sets of animals were used. The first study (cadmium dose effect) had three control dams and three cadmium treated dams and the second study (crossfoster study) had ten controls, ten cadmium treated dams, and four ethinyl estradiol treated dams. For the prepubertal time course study, the experiment had four control dams and four cadmium treated dams. For the prepubertal mammosphere studies, three to four sets of animals were used and each repeat had two to three control dams and two to three cadmium treated dams. In all studies, the experimental unit is the female offspring.

### 4.2. Morphological Analysis of the Mammary Gland

For whole mounts, the mammary glands processed as previously described [48]. Briefly, the glands were fixed in Carnoy’s fixative, defatted xylene, rehydrated, stained with carmine alum (Sigma), dehydrated in alcohol, and cleared in xylene. Prior to analysis, the samples were blinded. The digital images were obtained (MetaMorph Microscopy Automation & Image Analysis Software, Sunnyvale, CA, USA) and binarized. Density was calculated as the percentage of the epithelium relative to fat. Branching was calculated as the number of branching points per unit length along the two major mammary lactiferous ducts.

### 4.3. Mammosphere Culture

Mammospheres were isolated as previously described [48]. Briefly, whole mammary glands were digested with collagenase and hyaluronidase (Stemcell Technologies, Vancouver, British ColumbiaState, Canada; Gentle 10X Collagenase/Hyaluronidase #07919). Epithelial organoids were isolated and treated with trypsin-ethylenediaminetatraacetic acid (EDTA) (Stemcell Technologies), Dispase, and DNase I. To obtain a single cell suspension, the cell sample was diluted with cold Hanks’ Balanced Salt Solution Modified (Stemcell Technologies) supplemented with 2% FBS (HF) and filtered through a 40-μm cell strainer. The sample was then centrifuged and resuspended in serum-free mammary epithelial growth medium (MEGM, Lonza, Basal, Switzerland) supplemented with B-27 supplement minus vitamin A (2×; Invitrogen, Carlsbad, CA, USA), 20 ng/mL recombinant rat EGF (PeproTech, Rocky Hill, NJ, USA), 20 ng/mL recombinant rat bFGF (PeproTech), and 4 μg/mL heparin (Sigma). The cells were visualized and counted using a hemocytometer. In all experiments, the number of single cells was greater than 99%. Cells were plated in ultralow attachment 6-well plates (Corning, Corning, NY, USA) at a density of 40,000 cells in 2 mL of the supplemented MEGM per well and incubated in 5% CO_2_ incubator at 37 °C for 7–10 days. Although cell aggregates were present, only mammospheres larger than 60 μm in size (may have solid or hollow morphology) were counted and photo-documented. For mammosphere subcultures, the mammospheres were collected by centrifugation, dissociated with trypsin-EDTA, and triturated using a P-1000 pipette. Cold HF was added and the suspension was centrifuged. Supernatant was aspirated and the pellet resuspended in supplemented MEGM. Single cells were plated in ultralow attachment 6-well plates and incubated for 7–10 days as described above.

### 4.4. Real Time Reverse Transcriptase-Polymerase Chain Reaction

Details of the RT-qPCR assay are described elsewhere [48]. Briefly for RNA extraction, frozen tissue was pulverized in liquid nitrogen. Trizol reagent (Invitrogen) was added and the tissue was then homogenized. The homogenate was centrifuged and an equal volume of isopropanol was added to precipitate the RNA. The 260:280 ratio and concentration were determined. Samples were stored at −80 °C.

For the reverse transcriptase reaction, the RNA was first treated with deoxyribonuclease. For the RT reaction, Taqman RT Buffer, dNTPs, RNase inhibitor, MultiScribe reverse transcriptase (Applied Biosystems, Foster City, CA, USA, and RNA were incubated in the thermal cycler for 10 min at 25 °C, 30 min at 48 °C, and 5 min at 95 °C.

For the real-time polymerase chain reaction, each 10 μL reaction contained 5 μL of Sensimix II Probe Mastermix (Bioline, Cincinnati, OH, USA), 0.5 μL of 20× Assay on Demand (Applied Biosystems) and 4.5 μL of cDNA; or 5 μL of Sensimix SYBR (Bioline), 0.25 μL of 20 μM forward or reverse primer, and 4.5 μL of cDNA. For ERα, Aldh1, telomerase, Six1, keratin 15, keratin 8, Greb1, Areg, progesterone receptor, Arbp, and glyceraldehyde 3-phosphate dehydrogenase (GAPDH), the primer and probe sets were obtained from Applied Biosystems and spanned exon–exon boundaries. For the ERα 5′UTRs, the primer and probe sets were designed to span exon–exon boundaries and produce an amplicon between 100 and 200 bp. The sequence of the primers and probes and the size of the amplicon are as follows: For exon E1, forward E1: 5′-CTGCGCTGAGCCTCTTTTAAC-3′; reverse E1: CGGATGAGCCACCTGGAA, TaqMan^®^ probe: TCGGGCTCTACTCTT, and amplicon size 114 bp. For exon OT, forward OT: CGAGGCTTCCAGCAGGTTT, reverse OT: AGCCACGGGCTCTCCAA, TaqMan^®^ probe: CGATGTCTAAGAACAGGG, and amplicon size 149 bp. For exon O, forward O: CTACAAACCCATGGAACATTTCTG, reverse O: GGCTCAGCAGCGGATGA, TaqMan^®^ probe: CTTTTGAACCAGCAGGTGG, and amplicon size 167 bp. For exon ON, forward ON: TCATGACGCCATATTCCTCTACA, reverse ON: CAGCCGCCGAGGTACAGA, TaqMan^®^ probe: AGCCCTCTGCGTGCG, and amplicon size 145 bp. Samples were run on the 7900HT (Applied Biosystems) and the data analyzed by the 2^−ΔΔ*C*t^ method using the SDS 2.1 software (Applied Biosystems).

### 4.5. Nuclear Run-On Assay

The nuclear run-on assay is described in detail elsewhere [48]. Briefly, nuclei were prepared from frozen mammary glands and stored at −80 °C. For the run-on assay, nuclei were thawed on ice and incubated for 1 h at 26 °C in transcription buffer containing Biotin-16-UTP (Roche Diagnostics, Basal, Switzerland), ATP, CTP, GTP. The nuclei were collected by centrifugation, and the RNA was isolated using Trizol. Dynabeads M-280 Streptavidin (Invitrogen) were used to purify the nascent mRNA following the manufacturers protocol.

## 5. Conclusions

The results of this study show that *in utero* exposure to cadmium causes an expansion of the mammary stem/progenitor cell population and increases the expression of ERα. It is thought that mammary stem and/or progenitor cells are the targets of malignant transformation in the breast and overexpression of ERα is the first event in the development of ER positive breast cancers suggesting that *in utero* exposure to the metal predisposes the mammary gland to malignant transformation. The prevalence of cadmium in the environment and its ability to cross the placenta and accumulate in the mammary gland further suggests that early life exposure to cadmium contributes to the risk of developing breast cancer.
